# Paracrine Signaling Mediated by the Cytosolic Tryparedoxin Peroxidase of *Trypanosoma cruzi*

**DOI:** 10.3390/pathogens13010067

**Published:** 2024-01-10

**Authors:** María Laura Chiribao, Florencia Díaz-Viraqué, María Gabriela Libisch, Carlos Batthyány, Narcisa Cunha, Wanderley De Souza, Adriana Parodi-Talice, Carlos Robello

**Affiliations:** 1 Departamento de Bioquímica, Facultad de Medicina, Universidad de la República, Montevideo 11000, Uruguay; chiribao@fmed.edu.uy; 2 Laboratorio de Interacciones Hospedero–Patógeno—UBM, Institut Pasteur Montevideo, Montevideo 11000, Uruguay; florenciad@pasteur.edu.uy (F.D.-V.); gabilibisch@gmail.com (M.G.L.); 3 Laboratory of Vascular Biology and Drug Development, Institut Pasteur Montevideo, Montevideo 11000, Uruguay; batthyany@pasteur.edu.uy; 4 Instituto de Biofísica Carlos Chagas Filho, Centro Nacional de Biologia Estrutural e Bioimagem (CENABIO), Universidade Federal do Rio de Janeiro, Rio de Janeiro 21941-901, Brazil; narcisa@biof.ufrj.br (N.C.); wsouza@biof.ufrj.br (W.D.S.); 5 Sección Genética Evolutiva, Instituto de Biología, Facultad de Ciencias, Universidad de la República, Montevideo 11000, Uruguay

**Keywords:** peroxiredoxin, tryparedoxin peroxidase, Chagas disease, signaling, *Trypanosoma cruzi*

## Abstract

Peroxiredoxins are abundant and ubiquitous proteins that participate in different cellular functions, such as oxidant detoxification, protein folding, and intracellular signaling. Under different cellular conditions, peroxiredoxins can be secreted by different parasites, promoting the induction of immune responses in hosts. In this work, we demonstrated that the cytosolic tryparedoxin peroxidase of *Trypanosoma cruzi* (cTXNPx) is secreted by epimastigotes and trypomastigotes associated with extracellular vesicles and also as a vesicle-free protein. By confocal microscopy, we show that cTXNPx can enter host cells by an active mechanism both through vesicles and as a recombinant protein. Transcriptomic analysis revealed that cTXNPx induces endoplasmic reticulum stress and interleukin-8 expression in epithelial cells. This analysis also suggested alterations in cholesterol metabolism in cTXNPx-treated cells, which was confirmed by immunofluorescence showing the accumulation of LDL and the induction of LDL receptors in both epithelial cells and macrophages. BrdU incorporation assays and qPCR showed that cTXNPx has a mitogenic, proliferative, and proinflammatory effect on these cells in a dose–dependent manner. Importantly, we also demonstrated that cTXNPx acts as a paracrine virulence factor, increasing the susceptibility to infection in cTXNPx-pretreated epithelial cells by approximately 40%. Although the results presented in this work are from in vitro studies and likely underestimate the complexity of parasite–host interactions, our work suggests a relevant role for this protein in establishing infection.

## 1. Introduction

*Trypanosoma cruzi* is an obligate intracellular protozoan parasite with a complex life cycle characterized by different hosts and morphologically distinct stages. The epimastigote form is proliferative and found in the insect vector, whereas the amastigote and trypomastigote stages are the forms found in mammals, with the amastigote being the proliferative intracellular form. To establish a successful infection, the parasite must adhere to the mammalian cells and be internalized, a process mediated by various surface proteins [1]. During the life cycle, the parasite is exposed to various types of stress, one of the most important of which is oxidative stress mediated by the host’s professional phagocytic cells. Therefore, antioxidant systems play a fundamental role in the parasite’s survival in the mammalian host.

Typical peroxiredoxins (Prxs) are a highly conserved family of proteins whose main demonstrated intracellular role is a potent antioxidant function through their peroxidase activity in the presence of thioredoxin, thioredoxin reductase, and NADPH. These peroxidases do not have prosthetic groups. Instead, the functional unit of the protein is a dimer where each monomer provides one of the two conserved cysteines of the active site. Present in prokaryotes and eukaryotes, they are broadly distributed in intracellular compartments, such as the cytosol, mitochondria, peroxisome, endoplasmic reticulum, and plasma membrane [1,2,3]. As described decades ago [4], a dual role for Prx as a defender against oxidative stress and as a regulator of the H_2_O_2_ signaling pathway was proposed [5]. More recently, new functions have been described, giving them highly relevant physiological roles. Remarkably, some 2-Cys-Prxs act as chaperones, and the study of the cytosolic yeast 2-Cys Prxs, cPrx1, and cPrx2 has been extremely helpful in elucidating that, in addition to the reduction in hydroperoxides, a chaperone activity is associated with transitions from the oligomerization state [6,7].

Five distinct trypanothione-dependent peroxidases were found in *T. cruzi* parasites, with differences in subcellular locations and substrate specificity, namely two non-selenium containing glutathione peroxidases, an ascorbate-dependent haem peroxidase, and two tryparedoxin peroxidases (*Tc*TXNPxs): cTXNPx, located in the cytosol, and mTXNPx, located in mitochondria [8,9]. It has been shown in vitro that cTXNPx is a highly efficient enzyme in detoxifying oxidant molecules, such as H_2_O_2_ and ONOO^−^ [10]; on the other hand, this enzyme has also been shown to be able to prevent protein aggregation in vitro by acting as a molecular chaperone [11]. Both properties may contribute to its role as a virulence factor. Indeed, it has been shown that the most virulent strains have a higher expression level of this protein [12], and on the other hand, transfected parasites overexpressing cTXNPx have been shown to be more infective in vitro in both phagocytic and non-phagocytic cells [13] and also in vivo in a murine model [14].

In recent years, a newly identified feature of 2-Cys Prxs was their ability to be secreted. Initially observed in human lung cancer cells, the non-classical secretory pathway of Prx1 has been extensively studied, revealing that Prxs can be secreted in specific cells and conditions. Notably, Ridell and colleagues demonstrated that secreted Prx1 binds to TLR4, triggering the release of pro-inflammatory cytokines and suggesting a role in modulating macrophage immune responses [15]. These findings were replicated in various systems, highlighting a broader immune modulation role for mammalian Prxs. The significance of Prxs in immune modulation is evident in the brain post-stroke, where released peroxiredoxins act as potent danger signals, activating macrophages and inducing a detrimental cytokine response; remarkably, chaperone activity, rather than peroxidase activity, is crucial for this function [16].

In parasites, peroxiredoxins appear to play a crucial role in host–parasite interaction through immune modulation. A peroxiredoxin secreted by *F. hepatica* [17] induces the recruitment of M2 macrophages at the infection site, and recombinant fhPRX alone activates macrophages, promoting the secretion of IL4 and IL10. Similar ability was observed in homologous Prxs from *S. mansoni* and *H. contorts* [17]. In malaria, a recombinant peroxiredoxin induces innate immunity through TLR-4 with MyD-88 and MD-2 [18]. Remarkably, most proteomic studies of related parasite secretomes such as *Leishmania* spp. [19,20,21], *Trypanosoma brucei* [22,23], and *T. cruzi* [24,25,26] consistently identify at least one peroxiredoxin. Additionally, recombinant cTXNPx cytosolic peroxiredoxin of *T. cruzi* has the ability to recruit antigen-presenting cells in vivo and has also been demonstrated to be immunomodulatory in vivo, promoting a Th1 response in mice [27]. However, the secretion dynamics of this protein in the different stages of the parasite and how once secreted, it exerts its function at the local level and triggers a cellular response remain to be understood.

Epithelial cells constitute the first barrier against *T. cruzi*, actively participating in the innate immune response through the secretion of signaling molecules, like cytokines. The early response of HeLa cells to *T. cruzi* infection involves the induction of the chemokine IL8 and a disturbance of lipid metabolism [28], demonstrating a relevant role of these cells in the context of Chagas disease.

Given the relevance of secreted proteins in intercellular communication and the role of epithelial cells in the context of Chagas disease, in this work, we analyzed the secretion of cTXNPx by epimastigotes and trypomastigotes from *T. cruzi* and the interaction between this protein and epithelial cells. We demonstrated that the protein could generate a mitogenic and proinflammatory response in epithelial cells, acting as a paracrine virulence factor facilitating parasite invasion. The results obtained in this work point to cTXNPx as a relevant paracrine virulence factor in Chagas disease.

## 2. Materials and Methods

### 2.1. Cell and Parasite Culture

HeLa and Vero cell lines were maintained in Dulbecco’s Modified Eagle Medium (Gibco, Grand Island, NE, USA) supplemented with 10% fetal bovine serum (FBS) at 37 °C in a 5% CO_2_ atmosphere. Epimastigotes of the Dm28c strain were cultured in a liver infusion tryptose medium (LIT) supplemented with 10% fetal bovine serum (FBS). Cell-derived trypomastigotes were obtained from the infected monolayers of Vero cells(Vero CCL-81 cells were acquired from ATCC.).

### 2.2. Purification of Secreted Material from T. cruzi

For epimastigotes’ extracellular vesicles (eEVs) purification, parasites were grown to stationary phase in 10% SBF LIT. After washing with PBS, epimastigotes were resuspended in RPMI to a final concentration of 100 × 10 ^6^/mL for nutrient stress. After 24 h, parasites were pelleted by centrifugation at 2800× *g* for 15 min, and the supernatant was centrifuged at 15,000× *g* for 45 min for debris collection. The supernatant containing extracellular vesicles was ultracentrifuged at 100,000× *g* for 70 min, and the pellet was washed once with PBS. eEVs were resuspended in sterile PBS for transmission immunoelectron microscopy (TEM) analysis or in a Laemmly buffer 2X for Western blot studies. For trypomastigotes extracellular vesicle (tEV) purification, 10^8^ cell-derived trypomastigotes were incubated in PBS for 2 h, parasites were pelleted by centrifugation at 3000× *g* for 15 min, and the supernatant was treated as an epimastigotes supernatant. The vesicle-free fraction (VF) of epimastigotes was obtained from the supernatants of ultracentrifugation.

### 2.3. Transmission Electron Microscopy and Immunoelectron Microscopy

For negative staining, extracellular vesicles were adsorbed on nickel grids treated with Formvar 0.5% carbon for 20 min, and then washed in PBS and stained with 1% ammonium molybdate for 30 s and molybdate for 30 s. For immunolabeling, grids were fixed with 2% paraformaldehyde for 30 min at room temperature (RT) and then incubated with 50 mM NH4Cl in PBS for 5 min. Grids were blocked with 5% BSA in PBS for 10 min and permeabilized with 1% BSA 0.1% saponin in PBS for 5 min. The grids were incubated with an anti-cTXNPx antibody diluted in a blocking solution (1/200) for 30 min, washed, and incubated again with a diluted anti-cTXNPx antibody in PBS for 30 min. Finally, they were incubated with a 10 nm gold particle-conjugated anti-rabbit secondary antibody (Sigma-Aldrich, Burlington, MA, USA). After washing, the grids were visualized in a Zeiss EM 900 transmission electron microscope at the Centro Nacional de Biologia Estrutural e Bioimagem (CENABIO, Centro Nacional de Biologia Estrutural e Bioimagem (CENABIO), Federal University of Rio de Janeiro, UFRJ, RJ, Brazil.

### 2.4. Proteomics of Extracellular Vesicles from Epimastigotes

Vesicles isolated from epimastigotes were resuspended in 100 µL of water and purified with a “2D Clean-Up Kit” (GE Healthcare Life Sciences, Chicago, IL, USA). The pellet was then resuspended in 80 µL of bicarbonate buffer pH 8.0 with 2 M guanidine hydrochloride. The sample was reduced by adding 100 mM Dithiothreitol (DTT) and incubated at 56 °C for 1 h under agitation. After this period, the sample was alkylated by adding 15 µL of 300 mM iodoacetamide (IAM) and incubated for 45 min at RT in the dark. After pH control, the samples were digested with 1 µg trypsin for 18 h at 37 °C and injected into an LTQ Velos + ETD Mass Spectrometer (Thermo Fisher Scientific Inc., Waltham, MA, USA). Five different experiments were analyzed, of which four were independent biological replicates. Proteins were identified using the Mascot server (https://www.matrixscience.com accessed on 29 November 2023). Proteins identified in at least two different experiments were considered present.

### 2.5. Purification and Labeling of cTXNPx from E. coli Extracts

The clone pQE30-His6-cTXNPx or the empty vector pQE30 (Mock) in *E. coli* M15 cells [29] was grown at 37 °C with vigorous agitation in LB Broth containing 100 µg/mL ampicillin and 25 µg/mL kanamycin. Expression of recombinant His6-cTXNPx was induced with 0.5 mM isopropyl-b-D-thiogalactopyranoside (Euromedex) when the culture reached A600 = 0.6 and grown for an additional 4 h at 30 °C. Purification was performed as previously described [10]. Briefly, cells were harvested at 4000 g and resuspended in a binding buffer (50 mM sodium phosphate, pH 7.6, 10 mM imidazole, and 500 mM NaCl) and incubated with 1 mg/mL lysozyme (Sigma-Aldrich, Burlington, MA, USA) for 30 min on ice and sonicated. The supernatant was loaded into a 5 mL HiTrap affinity column (Amersham) charged with Ni^2+^ and equilibrated with a binding buffer at a 3 mL/min flow rate. After washing with a binding buffer, the His-tagged His6-cTXNPx was eluted in 50 mM sodium phosphate, pH 7.6, 400 mM imidazole, and 500 mM NaCl. Endotoxin levels were measured using a Pierce LAL Chromogenic Endotoxin Quantitation Kit (Thermo Fisher Scientific Inc., Waltham, MA, USA). Labeling of cTXNPx and mTXNPx was achieved with Atto 647n (Sigma) at pH 8.3, following the manufacturer’s instructions.

### 2.6. Binding Assays and Indirect Immunofluorescence (IIF)

For binding assays, 4 × 10^5^ HeLa cells were seeded on coverslips in 12-well plates and incubated with 1 µM Atto labeled cTXNPx or mTXNPx at 37 °C or 4 °C for 1 h in DMEM or purified secreted vesicles. After incubation, cells were washed twice with PBS, fixed with 4% paraformaldehyde in PBS for 20 min at RT, and blocked in 50 mM NH4Cl for 10 min. Cells were permeabilized with 0.5% triton in PBS for 10 min and blocked with 2% BSA in PBST for 30 min at RT. After washing with PBST, cells were incubated with anticTXNPx (1:2000), anti-tubulin antibodies diluted 1:1000 (Sigma), or anti-LAMP-1 (Cell Signaling Technology, Danvers, MA, USA) diluted 1:100 in 1% BSA-PBST for 1 h at RT. CellMask (Invitrogen, Carlsbad, CA, USA) diluted 1:1000 was used for membrane staining. After 3 washes with PBS 0.1% tween, cells were incubated with ALEXA 488 conjugated goat anti-rabbit or anti-mouse (Invitrogen) diluted 1:1000 for 1 h. After 3 washes, coverslips were mounted with a fluoroshield with DAPI (Sigma) and visualized with a Leica Confocal Microscope. For super-resolution microscopy, immunofluorescence was performed with the same protocol, but the images were obtained in a Zeiss Elyra PS.1 (emission and excitation wavelengths of 405, 488, and 642 nm), using the Super Resolution Structured Illumination Microscopy (SR-SIM) mode, which were subsequently solved using ZEISS software ZEN 2012 (version 9.1.1.5).

### 2.7. Protein Extracts and Western Blot

Protein extracts of HeLa cells were obtained by adding 150µL of a RIPA buffer supplemented with a protease inhibitor cocktail (Sigma), Nuclease Mix (GE Healthcare), and a PhosStop phosphatase inhibitor cocktail (Roche) directly to 1 × 10^6^ cells. Extracts (20 µg/lane) were subjected to 4–12% SDS-PAGE and electroblotted to a nitrocellulose membrane (GE Healthcare). Membranes were blocked with 5% non-fat milk in PBS containing 0.1% tween 20 (PBST) for 1 h at RT. After two washes with PBST, the following antibodies were used: anti-cTXNPx antisera 1:6000 diluted in 1% Bovine Seroalbumin BSA-PBST for 1 h at RT, anti-pERK, and anti-ERK (Cell Signaling) diluted 1:1000 in 5% BSA-PBST overnight at 4 °C. After 3 washes, membranes were incubated with HRP conjugated anti-rabbit diluted 1:7000 in BSA-PBST for 1 h at RT. The signal was developed with SuperSignal^TM^ West Pico Chemiluminescent Substrate (Thermo Fisher Scientific Inc., Waltham, MA, USA).

### 2.8. RNA-Seq Analysis

For RNA-seq analysis, 1µM cTXNPx was incubated with Hela cells (5 × 10^5^/well) in 6-well plates for 6 h. After washing with PBS, total RNA was isolated with phenol/chloroform, as described by the manufacturer (Tri Reagent, Sigma-Aldrich, Burlington, MA, USA). Processed samples were quantified in a Qubit (Invitrogen), exhibiting a high content of total RNA and a good quality without degradation, according to the RIN (RNA integrity number) values obtained from a Bioanalyzer 2100 (Agilent Technologies, Santa Clara, CA, USA), which were all above 8. Libraries were constructed using a ScriptSeq v2RNA-Seq library (Illumina, Foster City, CA, USA) kit and quantified with a Qubit dsDNA HS Assay kit (Invitrogen, Carlsbad, CA, USA). Sequencing was conducted using Illumina technology, and statistical analysis was performed in R. Read alignment with the human genome and read counts after filtering for poor quality scores, alignment, quantification, and differential expression analysis were performed in R Bioconductor using edgeR [30]. Genes with FDR values under 0.05 and a fold change value above 2 were considered differentially expressed. Gene ontology analysis was performed with Funrich software [31].

### 2.9. Proliferation Assay

To evaluate proliferation, HeLa cells were incubated with 0.2 or 1 μM cTXNPx for 2 h. Next, 50 μM bromodeoxyuridine (BrDU) (Sigma Aldrich) was added for an additional 4 h. Subsequently, the cells were fixed with 4% paraformaldehyde (PFA), and the IIF assay was performed as described above, using an anti-BrDU antibody diluted 1/50 and a secondary anti-mouse antibody conjugated to Alexa 488. The nuclei were stained with DAPI, and the images were processed using the Icy program [32]. For the analysis of BrdU incorporation, images were captured with an epifluorescence microscope, the cell nuclei were selected as regions of interest (ROIs), the mean fluorescence intensity (MFI) emitted at 488 nm was measured, and each cell was graphed as an independent point. As a positive control, the cells were incubated with a medium containing an FBS concentration of 20%.

### 2.10. RNA Extraction and Real-Time PCR

To evaluate cytokine expression in response to cTXNPx treatment, 1 × 10^6^ HeLa cells were plated and incubated for 1 h or 6 h with 0.2 µM or 1 µM cTXNPx, 1 µg/mL LPS (Sigma Aldrich), or a culture medium. After this period, the cells were washed and lysed with the Trireagent (Invitrogen).

RNA extraction was performed with a Direct-zol RNA miniprep kit (Zymo Research, Orange, CA, USA). cDNA was synthesized by reverse transcription using the M-MLV Reverse Transcriptase (Thermo Fisher Scientific Inc., Waltham, MA, USA) with oligo-dT primers, and 2500 ng of total RNA added as a template. cDNA was subjected to real-time using Sybr Green (KAPA SYBR^®^ FAST Universal 2X qPCR Master Mix, KapaBiosystems) as described [28]. PCR data were reported as the relative increase in mRNA transcripts in treated cells vs. control cells normalized by the respective levels of GAPDH mRNA, which was used as a housekeeping gene. The sequences of primers used are depicted in Supplementary Table S3.

### 2.11. LDL Receptor and LDL Uptake Quantification

LDLR expression and LDL uptake were quantified by immunofluorescence in a confocal microscope with an LDL uptake-based kit (Cayman Chemical, Ann Arbor, MI, USA) following the manufacturer’s instructions. Briefly, HeLa or J774 macrophage cells were seeded in 24-well plates and incubated with cTXNPx 1 µM or media for 6 h. After that, cells were washed with PBS and incubated with LDL-DyLight™550 for 6 h diluted 1:100 in serum-free DMEM for 45 min at 37 °C in the dark. Cells were washed 3 times with PBS and fixed with Cell-Based Assay Fixative. Immunofluorescence was carried out following the manufacturer’s recommendations. MFI was calculated using Image J software at an average of 80 cells per condition. Statistical analysis was performed on three biological replicates.

### 2.12. In Vitro Infection Assays

Semi-confluent HeLa cells (70%) in 12-well plates with coverslips were treated with 1 µM cTXNPx for 2 h or with media and then infected with cell-derived trypomastigotes at a ratio of 10 parasites per cell for 4 h in DMEM-10% FBS. After infection (t = 0 h), cells were washed twice with PBS, and the medium was replaced with fresh DMEM-10% FBS. Coverslips were removed at 0 and 48 h post-infection and fixed with 4% PFA for 20 min at RT. Cells were mounted with Fluoroshield containing DAPI (Sigma), and the number of infected cells and amastigotes per infected cell was determined by counting the nucleus of HeLa cells and the kinetoplast of amastigotes by epifluorescence microscopy. Analysis was performed using Icy software 2.4.2.0 [32].

### 2.13. Statistical Analysis

The statistical analyses were carried out in Prism software. Statistical evaluation was performed using Student’s *t*-test for unpaired data. Results were considered significant if *p* < 0.05. Data were expressed as means ± SEM.

## 3. Results

### 3.1. cTXNPx Is Secreted into the Culture Medium

Several studies have identified proteins secreted by *T. cruzi* [26,33,34,35], including tryparedoxin peroxidases. Given that cTXNPx is a cytosolic protein without a secretion signal sequence, we wondered whether release into the medium was associated with vesicles or exosomes. To this end, we purified extracellular vesicles (EVs) from epimastigotes by differential centrifugation and analyzed them by immunoelectron microscopy and Western blot; the vesicle-free fraction was also preserved for Western blotting analysis.

**Figure 1:**
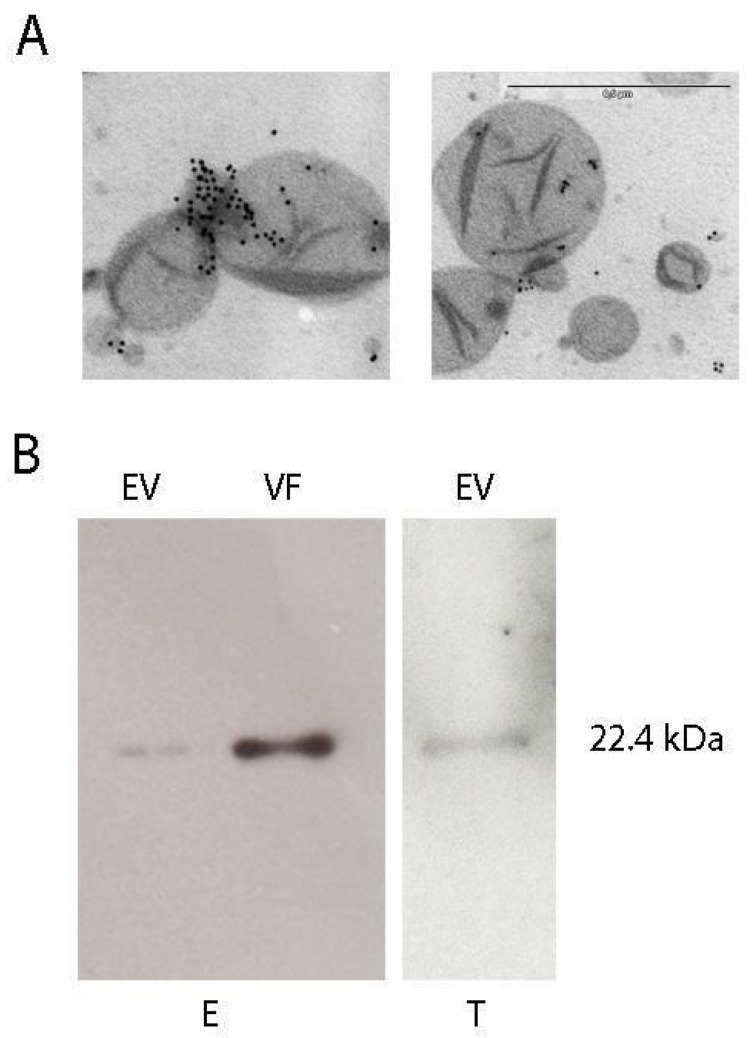
(**A**) Immunoelectron microscopy of extracellular vesicles purified from epimastigote supernatants (scale bar 0.5 μm). An anti-cTXNPx antibody was used at a dilution of 1/100. (**B**) Western blot using an anti cTXNPx antibody (1/5000) of extracellular vesicles and the free fraction of purified vesicles from epimastigote (E) or trypomastigote (T).

The size and shape of the vesicles secreted by epimastigotes were evaluated by transmission electron microscopy (TEM), which showed that they vary between approximately 100 and 500 nm. Immunolabeling with anti-cTXNPx antibodies revealed that the protein was present in vesicles of different sizes, suggesting that different secretion mechanisms are involved (Figure 1A). Western blot analysis of different secreted fractions with anti-cTXNPx antibodies showed that the protein was present in EVs released from epimastigotes and trypomastigotes, and the protein was also detected in the vesicle-free fraction (VF) of epimastigotes (Figure 1B). Proteomic analysis of the secreted vesicles was performed using mass spectrometry, and 27 different proteins were identified within the vesicles, including cTXNPx (Supplementary Table S1). Cellular component analysis of the secreted proteins showed a high enrichment of the proteasomal core complex and nucleosome compartments, and the ubiquitin-dependent protein catabolic process was one of the most represented biological processes in this group. In this context, the presence of gold labeling was observed not only within vesicles but also in proximity to electron-dense regions. Given that cTXNPx is a 20 nm decamer and can form high molecular weight oligomers, it is reasonable to conclude that these labeled spots may correspond to released proteins (Figure 1A).

### 3.2. cTXNPx Can Interact and Enter Mammalian Cells by Endocytosis

**Figure 2:**
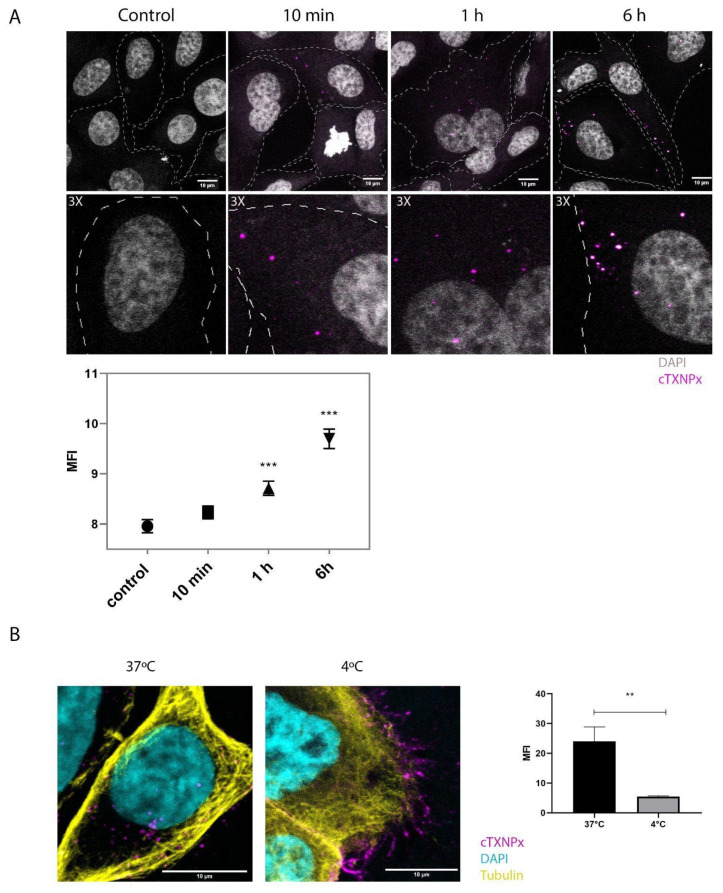
Interaction of cTXNPx with cells. (**A**) HeLa cells were incubated with epimastigote extracellular vesicles for 10 min, 1 h, or 6 h. Internalization was visualized by immunofluorescence with an anti-cTXNPx antibody, nuclei were stained with DAPI, and the dotted line represents the cell outline. The graph shows the mean fluorescence intensity of cTXNPx at the different incubation times and in the control. (**B**) HeLa cells were incubated with 1 µM recombinant Atto647-labeled cTXNPx for 1 h at 37 °C or 4 °C, the cytoskeleton was visualized with an anti-tubulin antibody, and the nuclei were stained with DAPI. The graph shows the mean fluorescence intensity of cTXNPx inside the cell at 37 °C and at 4 °C. (** *p* ≤ 0.01, *** *p* ≤ 0.001).

We subsequently investigated the ability of cTNPx to interact with and enter cells both as a free protein and associated with vesicles. We incubated epithelial cells with the purified vesicle fraction of epimastigotes (eEV) for 10 min, 1 h, and 6 h and evaluated the presence of cTXNPx by immunofluorescence. Figure 2A shows the cellular uptake of cTXNPx by epithelial cells facilitated through extracellular vesicles. Notably, a detectable presence of cTXNPx inside the cells becomes evident only after 10 min of interaction and increases significantly after 6 h. Interestingly, we observed that cTXNPx can be released from the free fraction of vesicles and enter epithelial cells (Supplementary Figure S1). This observation prompted an inquiry into whether the protein alone could interact with and traverse the epithelial cells. To address this, a recombinant cTXNPx was conjugated with Atto 647N fluorescent dye, enabling visualization; it was subsequently subjected to internalization assays conducted at temperatures of 37 °C and 4 °C. As depicted in Figure 2B, the results reveal that epithelial cells engage with cTXNPx at both examined temperatures. Specifically, at 37 °C, a conspicuous cellular internalization of the protein is noted. In stark contrast, interactions conducted at 4 °C solely manifested surface binding, with the fluorescence label conspicuously concentrated on the cell exterior. Taken together, these observations confirm the ability of the protein to effectively penetrate these epithelial cells at 37 °C, whereas interactions at 4 °C primarily retain the protein at the cell periphery. To assess the specificity of the input, we performed the same procedure with the mitochondrial peroxiredoxin mTXNPx, which we did not detect in the secreted fraction under our experimental conditions. The interaction assays showed that unlike cTXNPx, mitochondrial peroxiredoxin is barely internalized by epithelial cells under the same experimental conditions (Supplementary Figure S2).

**Figure 3:**
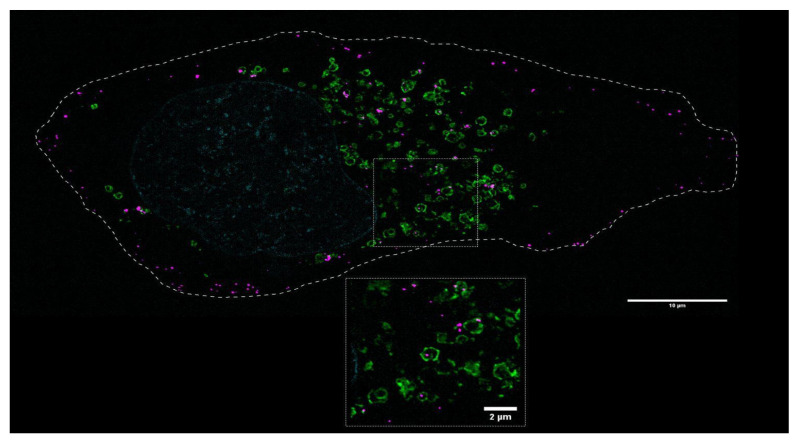
cTXNPx is present within lysosomal compartments. HeLa cells were incubated for 1 h with 1 µM Atto-labeled cTXNPx. Cells were stained with anti-LAMP1 antibodies and DAPI for lysosomal and nuclear staining, respectively, and visualized with a superresolution microscope. The image shows a representative Z stack, the scale bar in the main image is 10 µm, and in the inset is is 2 µm. A color map co-localization plugin showed a correlation index of 0.45. Green: LAMP1; Pink: cTXNPx. The dashed line represents the cell boundary.

Our findings support that the internalization of cTXNPx in epithelial cells is related to an energy-dependent mechanism, probably involving endocytosis. To confirm this hypothesis, we investigated the intracellular distribution of cTXNPx in relation to lysosomal structures using the LAMP-1 marker. Using super-resolution microscopy, we successfully visualized the presence of cTXNPx within lysosomal compartments. However, it is noteworthy that a fraction of the protein retains its superficial localization, indicating a distinct population of non-internalized protein entities (Figure 3).

### 3.3. cTXNPx Induces Unfolded Protein Response and Affects Cholesterol Metabolism in Epithelial Cells

To comprehensively investigate the effect of cTXNPx on epithelial cells, we performed a detailed RNA-seq analysis on HeLa cells after 6 h of incubation with the protein. Using this approach, we identified 64 differentially expressed genes (fold change > 2 and *p*-value < 0.05) in cells treated with cTXNPx, of which 53% were found to be overexpressed. Gene ontology analysis of the biological pathways represented by the upregulated genes revealed that unfolded protein response was significantly affected after incubation with cTXNPX (*p*-value < 0.05); other biological pathways, such as cholesterol biosynthesis and the activation of chaperones by ATF6-alpha, were also highlighted. Cellular compartment analysis showed that upregulated genes are linked to the endoplasmic reticulum, the Golgi apparatus, and the plasma membrane, suggesting that endocytosis/exocytosis is also involved (Table 1 and Supplementary Table S2). Analysis of transcription factor enrichment in upregulated genes showed a predominance of NFYA (58.62%), SP1 (65.52%), SP4 (44.83%), and EGR1 (38%) (Table 1), which are involved in many processes such as mitogenesis, cell growth, immune responses, and steroid metabolism, among others. On the other hand, downregulated genes are enriched in transcription factors that regulate cell differentiation (CUX1 and MYF5).

### 3.4. cTXNPx Promotes ERK Activation and Cell Proliferation

**Figure 4:**
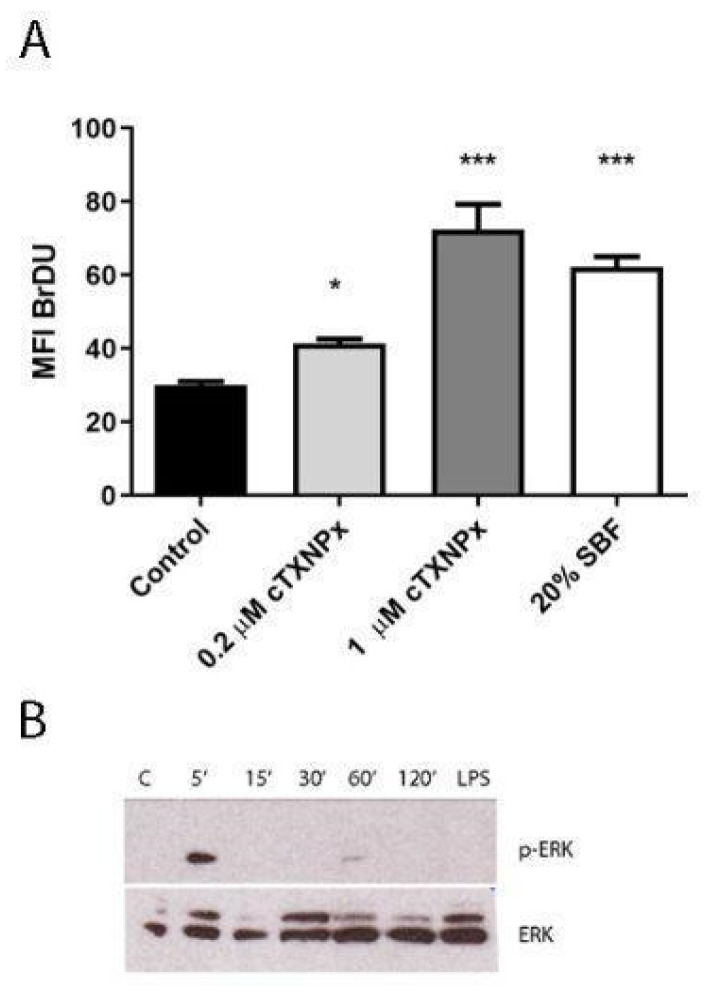
cTXNPx induces cell proliferation and activates the ERK pathway. (**A**) Incorporation of BrdU in cTXNPx-treated epithelial cells. (**B**) Western blot on epithelial cell extracts treated with cTXNPx for 5, 15, 30, 60, and 120 min. LPS was used as an endotoxin control (* *p* ≤ 0.05, *** *p*≤ 0.001).

RNA-seq data revealed that treatment of epithelial cells with cTXNPx induces the upregulation of genes related to the NFYA transcription factor (TMEM50B; GLRX; PIM2; SGK1; HOOK1), which is involved in the regulation of proliferation and lipid metabolism [34]. In addition, since it is known that *T. cruzi* infection also induces proliferation in different cell lines [35,36,37], we wondered whether cTXNPx could have a mitogenic effect on epithelial cells. To this purpose, we examined the proliferation of HeLa cells by measuring the incorporation of Bromodeoxyuridine (BrdU) after pretreatment with cTXNPx, using 20% fetal bovine serum as a positive proliferation control. As depicted in Figure 4A, the pretreatment of HeLa cells with 1 µM cTXNPx resulted in a significant increase in BrdU incorporation of approximately 30%, whereas the effect was negligible for the 0.2 µM concentration. Given the central role of the MAPK pathway in cell proliferation, we further investigated the activation of ERK1/2, a critical component of the MAPK pathway, through Western blot analysis after treatment with cTXNPx. As illustrated in Figure 4B, treatment of HeLa cells with cTXNPx leads to the phosphorylation of ERK. Kinetic analysis reveals a peak in phosphorylation occurring 5 min post-interaction, indicating that parasite peroxiredoxin can promptly activate the ERK pathway upon contact with HeLa cells. No activation of the pathway was observed using LPS as a control. This observation suggests a potential role for parasite peroxiredoxin in promoting cell proliferation. Remarkably, an increase in host cell proliferation could potentially benefit parasite invasion, a phenomenon observed in certain intracellular parasites [38].

### 3.5. Induction of Inflammatory Cytokines after cTXNPx Treatment

**Figure 5:**
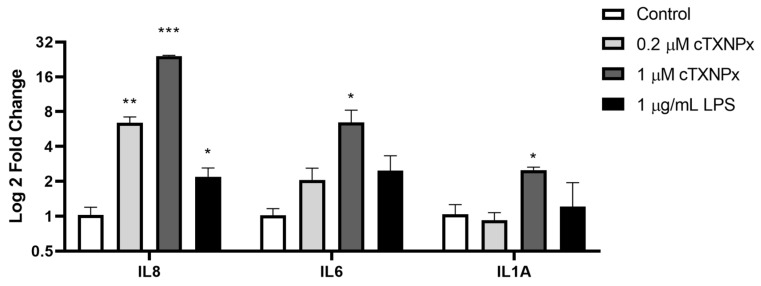
Quantification of IL1A, IL6, and IL1A expression by qPCR in HeLa cells after 6 h of interaction with cTXNPx 1 µM or LPS. Quantification was performed with ΔΔt Ct using GAPDH as a housekeeping gene (* *p* ≤ 0.05, ** *p* ≤ 0.01, *** *p*≤ 0.001).

RNA-seq analysis also revealed that cTXNPx induces the expression of genes related to inflammation and immune responses, like IL8 (114-fold induction) and PTX3 (31-fold induction, Supplementary Figure S2. In addition, previous reports have shown that peroxiredoxins have immunomodulatory properties and can induce cytokine expression and secretion [15,16,17,39,40], and in particular, we have shown that cTXNPx induces a Th1 response in vivo [27]. To assess the effect of cTXNPx on cytokine expression in epithelial cells, we performed quantitative PCR (qPCR) to measure the levels of IL8, IL6, and IL1A. Our results showed that the protein stimulates the IL8 and IL6 overexpression and, to a lesser extent, IL1A (as depicted in Figure 5). cTXNPx induced IL8 expression dose–dependently, while LPS was inefficient in inducing cytokines in this cell line (Figure 5).

### 3.6. cTXNPx Induces LDL Receptor Expression and Uptake of LDL in Epithelial Cells and Macrophages

**Figure 6:**
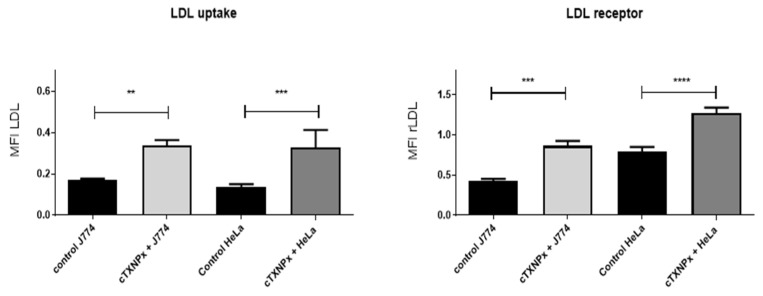
Quantification of rLDL expression and LDL uptake of HeLa and J774 cells after 6 h of incubation with 1 µM cTXNPx. The mean fluorescence intensity (MFI) at 550 (for LDL uptake) and 480 nm (for rLDL expression) was quantified with Fiji software. The graph shows a representative result of three biological replicates performed (** *p* ≤ 0.05, *** *p* ≤ 0.01, **** *p*≤ 0.001).

As previously indicated, RNAseq analysis uncovered significant changes in cholesterol biosynthesis and transport pathways following treatment with cTXNPx. This treatment led to a remarkable five-fold upregulation of the LDLR gene. In this context, we quantitatively evaluated the expression of the LDL receptor and the uptake of LDL. Both HeLa cells and macrophages treated with 1 µM recombinant cTXNPx for 6 h showed an increase both in LDL receptor expression and in the intracellular accumulation of fluorescent LDL (Figure 6 and Supplementary Figure S3).

We also demonstrated the endocytosis of cTXNPx by murine macrophages, and given the relevance of cholesterol uptake for foam cell formation, we set out to evaluate cholesterol uptake in a murine macrophage line. As shown in Figure 6, macrophages treated with cTXNPx also induce the expression of the LDL receptor, as well as the incorporation of LDL.

This occurrence holds significant importance since it is well established that *T. cruzi* uses this receptor [39], as well as cholesterol-rich microdomains, for invasion [40]. Therefore, it is plausible that secreted cTXNPx directly favors parasite infection through these mechanisms.

### 3.7. cTXNPx Promotes T. cruzi Entry into HeLa Cells

**Figure 7:**
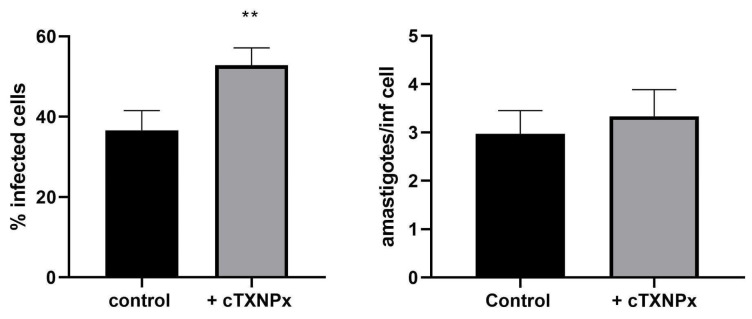
Evaluation of the invasive and infective capacity of trypomastigotes to HeLa cells pre-incubated with 1 µM cTXNPx for 2 h. The percentage of infected cells (**left**) after 4 h of parasite–cell interaction (t = 0 h post-infection) and the number of amastigotes per infected cell (**right**) at 48 h post-infection were evaluated by microscopy. The graphs show the results of three independent biological replicates (** *p* ≤ 0.01).

The observed increase in LDL receptor expression, along with other effects induced by cTXNPx treatment in HeLa cells, led to the hypothesis that the interaction between the parasite’s peroxiredoxin and host cells might enhance parasite infectivity. To test this hypothesis, we conducted a thorough analysis of infection rates in cTXNPx-treated cells compared to control cells. Our results show that the pretreatment of epithelial cells with 1µM of cTXNPx increases the percentage of infected cells by approximately 44%. On the other hand, the number of amastigotes per infected cell at 48 h also showed an increase. However, this difference was not statistically significant. This result suggests a specific role of cTXNPx in enhancing the initial stages of parasite–host cell interaction, leading to increased infectivity (Figure 7).

## 4. Discussion

Communication between parasites and host cells through released molecules has emerged as one of the most critical mechanisms of infectivity and persistence. Several parasites release vesicles containing virulence factors [41,42,43,44] and free proteins, which can interact with their hosts. In *T. cruzi*, secreted transialidases [45,46,47] and cruzipain [48,49] can modulate immune response and apoptosis in host cells, contributing to Chagas pathogenesis. In this work, we show that the cytosolic peroxiredoxin of *T. cruzi* is released by the parasite both in vesicles and in soluble form in epimastigotes and trypomastigotes. Peroxiredoxins were detected by proteomic approaches in the secretomes of other parasites and the proteome of *T. cruzi*-secreted vesicles [33,50]. These antioxidant proteins are also involved in other cellular functions such as inflammation, apoptosis, chaperones, and the regulation of metabolism [51,52]. We show that released cTXNPx can enter epithelial cells via the endocytic pathway, reach lysosomes, and alter cellular functions.

Transcriptomic analysis of cTXNPx-treated epithelial cells revealed that 64 genes changed their expression, half of which were upregulated during treatment (Supplementary Table S2). Gene ontology analysis of the significant biological pathways affected by cTXNPx suggests that the protein causes disturbances in cellular homeostasis, causing endoplasmic reticulum stress and alterations in cholesterol metabolism. Other altered biological processes are related to immune response and proliferation.

*T. cruzi* infection has been associated with altering cell cycle control and cell proliferation in different models [28,35,36,37]. Here, we found that cTXNPx can promote cell proliferation and MAPK ERK1/2 activation. The extracellular signal-related kinases (ERK1/2) are associated with many cellular processes, such as cell proliferation, differentiation, migration, senescence, and apoptosis [53]. It is also known that altering the extracellular redox environment is sufficient to induce MAPK phosphorylation, and this effect is mediated, at least in part, by extracellular thiols and TGF-beta signaling [54].

In epithelial cells, the activation of the MAPK pathway by cTXNPx during infection could promote host cell survival, facilitate parasite dissemination, and influence the development of Chagas cardiomyopathy, particularly in cardiomyocytes and fibroblasts. In some intracellular pathogens, the induction of S-phase and cell proliferation is advantageous for invasion [38]. This work shows that incubating epithelial cells with cTNXPx increases cell proliferation, possibly mediated by ERK signaling.

Although it is unclear how cTXNPx induces ER stress, the unfolded protein response has been shown to function as a defense mechanism against intracellular pathogen infection [55]. We hypothesize that ER stress can be triggered by intracellular cholesterol perturbations and a redox imbalance. ERK activation in response to ER stress is a mechanism that prevents apoptosis [56]; in our model, ERK activation may be a consequence of ER stress activation. Cholesterol accumulation seems to be a common cellular response to intracellular pathogens such as viruses [57,58], bacteria [59,60,61], and protozoa [28,62,63,64,65]. This molecule plays many roles in host–parasite interaction. First, lipid rafts and cholesterol-rich microdomains are relevant for the internalization of some pathogens [66,67,68,69], probably because these microdomains are enriched in caveolin, flotillin, PI3K, p85, and other proteins involved in infection [67]. Second, cholesterol accumulation and inflammation are predisposed to cardiovascular diseases, such as atherosclerosis [70]. Finally, intracellular cholesterol could serve as a lipid source for intracellular pathogens, e.g., for membrane synthesis during replication [71,72]. In *T. cruzi* amastigotes, it has been shown that 80% of the membrane sterols are cholesterol, probably derived from the host [68], which is in contrast to epimastigotes, where ergosterol is the principal sterol [73]. In this regard, we have shown that intracellular LDL accumulation occurs in both epithelial cells and macrophages in vitro, and this is coupled with the inflammatory context in response to cTXNPx. Some reports propose a link between Chagas disease and atherosclerosis. One of them shows that *T. cruzi*-infected mice fed a Western diet have a higher risk of developing atherosclerosis [71].

Since the LDL receptor has been implicated in *T. cruzi* invasion [39] and treatment of HeLa with cTXNPx increases LDL receptor expression and LDL uptake, we asked whether cTXNPx could affect cell susceptibility to infection. Our results show that pre-incubation with recombinant cTXNPx increases the susceptibility of HeLa cells to *T. cruzi* infection. This increase in susceptibility is related to the invasion process, increasing the percentage of infected cells.

Another result was the induction of cytokines in epithelial cells mediated by cTXNPx, particularly IL8. It is remarkable that this cytokine—a chemotactic factor for neutrophils and angiogenic molecules—is the most upregulated gene in the early response to *T. cruzi* infection in HeLa cells [28]. In this work, we describe the protein responsible for this response, which can be essential for the dissemination, invasion, survival, and persistence of *T. cruzi* in its host. Levels of IL8 and IL32 were also associated with disease control in fibroblasts infected with two different strains of *T. cruzi*, suggesting their relevance in the pathogenesis of Chagas disease [72]. One of the most relevant and poorly understood aspects of Chagas disease is that it exhibits significant variability in infection outcomes, influenced by factors such as parasite strain, virulence, and individual susceptibility. One of the chronic manifestations is Chagas heart disease, an inflammatory cardiomyopathy. Many hypotheses have been proposed to explain how the infection promotes tissue inflammation, but the mechanism is not fully understood. One hypothesis is that some factors of the parasite (but not the parasite) are responsible for this response, for example, secreted factors. Another hypothesis for the pathogenesis of Chagas disease is autoimmunity, initiated by cell damage and antigen release or by molecular mimicry between parasite epitopes and host components [73,74]. In this work, we show that *T. cruzi* cTXNPx is secreted by epimastigotes and trypomastigotes by different non-classical mechanisms and that the protein is found in extracellular vesicles and is also soluble in a vesicle-free fraction. Secreted cTXNPx becomes relevant in both aspects of the Chagas pathogenesis mechanisms. On the one hand, cTXNPx induces an immune response in epithelial cells and the release of proinflammatory cytokines by epithelial cells in vitro, mainly IL8, IL6, and IL1A; in fact, the protein was shown to have an immunomodulatory role in mice, recruiting antigen-presenting cells and inducing a Th1 response [27]. On the other hand, its similarity to human peroxiredoxins (70% amino acid identity) suggests a potential for autoimmune responses. The released cTXNPx may act as a danger signal (DAMP), promoting inflammation and tissue damage in the context of infection.

Other proteins, particularly chaperones, have been implicated in host modulation. Chlamydial Hsp60, a protein that can be secreted, can induce proliferation in endothelial cells through TLR4 and ERK activation [75]. Interestingly, many peroxiredoxins have been characterized as chaperones [6,52,76,77].

The cTXNPx protein has already been pointed out as a virulence factor for *T. cruzi* since parasites overexpressing this enzyme are more infective [13,14], attributing this characteristic to its antioxidant function. In this work, we demonstrated that cTXNPx is secreted by different stages of the parasite being able to interact with mammalian cells by increasing susceptibility to infection, i.e., acting as a paracrine virulence factor. This effect seems to be independent of its antioxidant capacity since the other proteins that are part of the antioxidant system would not be acting under these conditions.

Some studies have investigated the effect of microvesicles on parasites (differentiation) and/or on host cells, showing that microvesicles derived from *T. cruzi* influence their susceptibility to infection [42] and affect gene expression [78]. A pro-inflammatory role of vesicles has been demonstrated both in vitro [78] and in vivo [41]. However, evaluating the effect of microvesicles on epithelial cells does not allow us to dissect the effect of the protein of interest; therefore, we decided to study the effect of recombinant cTXNPx. Notably, the response observed with cTXNPx in our model is consistent with the results reported using secreted vesicles from *T. cruzi*. It would be interesting to evaluate whether the effect of cTXNPx delivered as vesicles is the same as the recombinant protein.

This study is an approximation to understanding the signaling function of an isolated and recombinantly expressed molecule and, therefore, has some limitations. On the one hand, secretion in vesicles implies an association of cTXNPx with other proteins or molecules present that may modulate the observed response. On the other hand, expression in heterologous systems may also have some disadvantages in terms of preservation of post-translational modifications and the presence of contaminants. In this sense, the protocol used has been described in several publications, where it has been shown that the protein retains its functionality and oligomerization capacity [10,11,27,29], and an additional endotoxin removal step has been added to further improve purity. We believe that this work contributes to the knowledge of the signaling function of a very abundant and actively secreted protein, which has already been shown to be an important virulence factor for the parasite. The generation of knockout lines would be a very useful tool in this sense to complement the results presented in this work.

In conclusion, our study reveals the secretion of the *T. cruzi* cytosolic peroxiredoxin, cTXNPx, in two parasite stages, together with its interaction and internalization by epithelial cells. We showed that this protein disrupts protein folding, leading to endoplasmic reticulum (ER) stress, stimulates proliferation, probably through ERK activation, and induces the expression of inflammatory cytokines, particularly IL8. In addition, the secreted peroxiredoxin serves as a paracrine virulence factor, enhancing infectivity by increasing cell susceptibility. The combined mitogenic effects, the production of inflammatory mediators, such as IL8 and IL6, and the ability to facilitate infection underscore the important role of cTXNPx in the pathogenesis of Chagas disease.

## Figures and Tables

**Table 1:** Gene ontology analysis of upregulated genes in epithelial cells treated with cTXNPx. GO analysis was performed with Funrich (functional analysis enrichment tool) [33], and the table shows the biological pathway, cellular component, and transcription factor enrichment analysis.

**Biological pathway**	**% of Genes**	**F.E.**	***p*-value**
Unfolded protein response	30.8	30.8	6.11 × 10^−6^
Activation of chaperones by ATF6-alpha	15.4	107.9	1.41 × 10^−4^
PERK-regulated gene expression	15.4	88.3	2.15 × 10^−4^
Cholesterol biosynthesis	15.4	48.6	7.34 × 10^−4^
Ascorbate recycling (cytosolic)	7.7	483.5	2.07 × 10^−3^
Activation of chaperones by IRE1-alpha	15.4	21.6	3.71 × 10^−3^
LDL-mediated lipid transport	7.7	121.8	8.24 × 10^−3^
ER Quality Control Compartment (ERQC)	7.7	81.2	1.23 × 10^−2^
Metabolism of lipids and lipoproteins	23.1	5.7	1.42 × 10^−2^
Calnexin/calreticulin cycle	7.7	40.7	2.45 × 10^−2^
**Cellular component**	**% of genes**	**F.E.**	***p*-value**
Endoplasmic reticulum	40.0	5.3	8.19 × 10^−7^
ER-Golgi intermediate compartment	10.0	48.6	3.09 × 10^−5^
Integral to endoplasmic reticulum membrane	6.7	31.4	1.84 × 10^−3^
Microsome	10.0	9.4	3.97 × 10^−3^
Clathrin-coated endocytic vesicle membrane	3.3	243.7	4.12 × 10^−3^
Lysosome	30.0	2.7	4.17 × 10^−3^
Extrinsic to the external side of the plasma membrane	3.3	162.7	6.17 × 10^−3^
Low-density lipoprotein particle	3.3	81.5	1.23 × 10^−2^
Perinuclear region of cytoplasm	6.7	8.2	2.48 × 10^−2^
**Transcription factor**	**% of genes**	**F.E.**	***p*-value**
NFYA	58.6	3.5	3.87 × 10^−7^
HNF4A	37.9	1.9	1.80 × 10^−2^
LMO2	13.8	3.5	2.72 × 10^−2^
EGR1	37.9	1.8	2.83 × 10^−2^
ZNF143	13.8	3.4	2.84 × 10^−2^
SP1	65.5	1.4	3.31 × 10^−2^
ZEB1	10.3	3.9	3.98 × 10^−2^
ESR1	13.8	3.0	4.13 × 10^−2^
SP4	44.8	1.6	4.44 × 10^−2^

## Data Availability

Data are contained within the article.

## Supplementary Materials

The following supporting information can be downloaded at: https://www.mdpi.com/article/10.3390/pathogens13010067/s1, Figure S1: Incorporation of cTXNPx from the vesicle-free fraction; Figure S2: Interaction of recombinant mTXNPx with HeLa cells; Figure S3: Expression of LDL receptor and internalization of human LDL; Table S1: List of proteins identified in secreted vesicles from epimastigotes by mass spectrometry analysis; Table S2: List of differentially expressed genes in RNAseq analysis; Table S3: List of primers used in qPCR experiments.

## References

[1] De Souza W. (2014). *Trypanosoma cruzi*-Host Cell Interaction. Front. Immunol..

[2] Hall A., Karplus P.A., Poole L.B. (2009). Typical 2-Cys peroxiredoxins--structures, mechanisms and functions. FEBS J..

[3] Wood Z.A., Poole L.B., Karplus P.A. (2003). Peroxiredoxin evolution and the regulation of hydrogen peroxide signaling. Science.

[4] Chae H.Z., Kim I.H., Kim K., Rhee S.G. (1993). Cloning, sequencing, and mutation of thiol-specific antioxidant gene of Saccharomyces cerevisiae. J. Biol. Chem..

[5] Kang S.W., Rhee S.G., Chang T.-S., Jeong W., Choi M.H. (2005). 2-Cys peroxiredoxin function in intracellular signal transduction: Therapeutic implications. Trends Mol. Med..

[6] Jang H.H., Lee K.O., Chi Y.H., Jung B.G., Park S.K., Park J.H., Lee J.R., Lee S.S., Moon J.C., Yun J.W. (2004). Two enzymes in one; two yeast peroxiredoxins display oxidative stress-dependent switching from a peroxidase to a molecular chaperone function. Cell.

[7] Moon J.C., Hah Y.S., Kim W.Y., Jung B.G., Jang H.H. (2005). Oxidative Stress-dependent Structural and Functional Switching of a Human 2-Cys Peroxiredoxin Isotype II That Enhances HeLa Cell Resistance to H_2_O_2_-induced Cell Death. J. Biol. Chem..

[8] Piñeyro M.D., Arias D., Parodi-Talice A., Guerrero S., Robello C. (2021). Trypanothione Metabolism as Drug Target for Trypanosomatids. Curr. Pharm. Des..

[9] Wilkinson S.R., Temperton N.J., Mondragon A., Kelly J.M. (2000). Distinct mitochondrial and cytosolic enzymes mediate trypanothione-dependent peroxide metabolism in *Trypanosoma cruzi*. J. Biol. Chem..

[10] Piñeyro M.D., Arcari T., Robello C., Radi R., Trujillo M. (2011). Tryparedoxin peroxidases from *Trypanosoma cruzi*: High efficiency in the catalytic elimination of hydrogen peroxide and peroxynitrite. Arch. Biochem. Biophys..

[11] Piñeyro M.D., Arias D., Ricciardi A., Robello C., Parodi-Talice A. (2019). Oligomerization dynamics and functionality of *Trypanosoma cruzi* cytosolic tryparedoxin peroxidase as peroxidase and molecular chaperone. Biochim. Biophys. Acta Gen. Subj..

[12] Zago M.P., Hosakote Y.M., Koo S.-J., Dhiman M., Piñeyro M.D., Parodi-Talice A., Basombrio M.A., Robello C., Garg N.J. (2016). TcI Isolates of *Trypanosoma cruzi* Exploit the Antioxidant Network for Enhanced Intracellular Survival in Macrophages and Virulence in Mice. Infect. Immun..

[13] Piñeyro M.D., Parodi-Talice A., Arcari T., Robello C. (2008). Peroxiredoxins from *Trypanosoma cruzi*: Virulence factors and drug targets for treatment of Chagas disease?. Gene.

[14] Piacenza L., Peluffo G., Alvarez M.N., Kelly J.M., Wilkinson S.R., Radi R. (2008). Peroxiredoxins play a major role in protecting *Trypanosoma cruzi* against macrophage- and endogenously-derived peroxynitrite. Biochem. J..

[15] Riddell J.R., Wang X.-Y., Minderman H., Gollnick S.O. (2010). Peroxiredoxin 1 stimulates secretion of proinflammatory cytokines by binding to TLR4. J. Immunol..

[16] Shichita T., Hasegawa E., Kimura A., Morita R., Sakaguchi R., Takada I., Sekiya T., Ooboshi H., Kitazono T., Yanagawa T. (2012). Peroxiredoxin family proteins are key initiators of post-ischemic inflammation in the brain. Nat. Med..

[17] Donnelly S., Stack C.M., O’Neill S.M., Sayed A.A., Williams D.L., Dalton J.P. (2008). Helminth 2-Cys peroxiredoxin drives Th2 responses through a mechanism involving alternatively activated macrophages. FASEB J..

[18] Furuta T., Imajo-Ohmi S., Fukuda H., Kano S., Miyake K., Watanabe N. (2008). Mast Cell-Mediated Immune Responses through IgE Antibody and Toll-like Receptor 4 by Malarial Peroxiredoxin. Eur. J. Immunol..

[19] Silverman J.M., Chan S.K., Robinson D.P., Dwyer D.M., Nandan D., Foster L.J., Reiner N.E. (2008). Proteomic analysis of the secretome of *Leishmania donovani*. Genome Biol..

[20] Pissarra J., Pagniez J., Petitdidier E., Séveno M., Vigy O., Bras-Gonçalves R., Lemesre J.-L., Holzmuller P. (2022). Proteomic Analysis of the Promastigote Secretome of Seven Leishmania Species. J. Proteome Res..

[21] Silverman J.M., Clos J., De’Oliveira C.C., Shirvani O., Fang Y., Wang C., Foster L.J., Reiner N.E. (2010). An exosome-based secretion pathway is responsible for protein export from Leishmania and communication with macrophages. J. Cell Sci..

[22] Nten C.M.A., Sommerer N., Rofidal V., Hirtz C., Rossignol M., Cuny G., Peltier J.-B., Geiger A. (2010). Excreted/secreted proteins from trypanosome procyclic strains. J. Biomed. Biotechnol..

[23] Geiger A., Hirtz C., Bécue T., Bellard E., Centeno D., Gargani D., Rossignol M., Cuny G., Peltier J.-B. (2010). Exocytosis and protein secretion in Trypanosoma. BMC Microbiol..

[24] Bayer-Santos E., Lima F.M., Ruiz J.C., Almeida I.C., da Silveira J.F. (2014). Characterization of the small RNA content of *Trypanosoma cruzi* extracellular vesicles. Mol. Biochem. Parasitol..

[25] Brossas J.-Y., Gulin J.E.N., Bisio M.M.C., Chapelle M., Marinach-Patrice C., Bordessoules M., Palazon Ruiz G., Vion J., Paris L., Altcheh J. (2017). Secretome analysis of *Trypanosoma cruzi* by proteomics studies. PLoS ONE.

[26] Queiroz R.M.L., Ricart C.A.O., Machado M.O., Bastos I.M.D., de Santana J.M., de Sousa M.V., Roepstorff P., Charneau S. (2016). Insight into the Exoproteome of the Tissue-Derived Trypomastigote form of *Trypanosoma cruzi*. Front. Chem..

[27] López L., Chiribao M.L., Girard M.C., Gómez K.A., Carasi P., Fernandez M., Hernandez Y., Robello C., Freire T., Piñeyro M.D. (2021). The cytosolic tryparedoxin peroxidase from *Trypanosoma cruzi* induces a pro-inflammatory Th1 immune response in a peroxidatic cysteine-dependent manner. Immunology.

[28] Chiribao M.L., Libisch G., Parodi-Talice A., Robello C. (2014). Early *Trypanosoma cruzi* infection reprograms human epithelial cells. Biomed. Res. Int..

[29] Piñeyro M.D., Pizarro J.C., Lema F., Pritsch O., Cayota A., Bentley G.A., Robello C. (2005). Crystal structure of the tryparedoxin peroxidase from the human parasite *Trypanosoma cruzi*. J. Struct. Biol..

[30] Robinson M.D., McCarthy D.J., Smyth G.K. (2010). edgeR: A Bioconductor package for differential expression analysis of digital gene expression data. Bioinformatics.

[31] Fonseka P., Pathan M., Chitti S.V., Kang T., Mathivanan S. (2021). FunRich enables enrichment analysis of OMICs datasets. J. Mol. Biol..

[32] de Chaumont F., Dallongeville S., Chenouard N., Hervé N., Pop S., Provoost T., Meas-Yedid V., Pankajakshan P., Lecomte T., Le Montagner Y. (2012). Icy: An open bioimage informatics platform for extended reproducible research. Nat. Methods.

[33] de Chaumont F., Dallongeville S., Chenouard N., Hervé N., Pop S., Provoost T., Meas-Yedid V., Pankajakshan P., Lecomte T., Le Montagner Y. (2013). Proteomic analysis of *Trypanosoma cruzi* secretome: Characterization of two populations of extracellular vesicles and soluble proteins. J. Proteome Res..

[34] de Chaumont F., Dallongeville S., Chenouard N., Hervé N., Pop S., Provoost T., Meas-Yedid V., Pankajakshan P., Lecomte T., Le Montagner Y. (2023). NFYA promotes malignant behavior of triple-negative breast cancer in mice through the regulation of lipid metabolism. Commun. Biol..

[35] Costales J.A., Daily J.P., Burleigh B.A. (2009). Cytokine-dependent and–independent gene expression changes and cell cycle block revealed in *Trypanosoma cruzi*-infected host cells by comparative mRNA profiling. BMC Genom..

[36] Goldenberg R.C.d.S., Iacobas D.A., Iacobas S., Rocha L.L., Fortes F.d.S.d.A., Vairo L., Nagajyothi F., de Carvalho A.C.C., Tanowitz H.B., Spray D.C. (2009). Transcriptomic alterations in *Trypanosoma cruzi*-infected cardiac myocytes. Microbes Infect..

[37] Li Y., Shah-Simpson S., Okrah K., Belew A.T., Choi J., Caradonna K.L., Padmanabhan P., Ndegwa D.M., Temanni M.R., Bravo H.C. (2016). Transcriptome Remodeling in *Trypanosoma cruzi* and Human Cells during Intracellular Infection. PLoS Pathog..

[38] Lavine M.D., Arrizabalaga G. (2009). Induction of mitotic S-phase of host and neighboring cells by Toxoplasma gondii enhances parasite invasion. Mol. Biochem. Parasitol..

[39] Nagajyothi F., Weiss L.M., Silver D.L., Desruisseaux M.S., Scherer P.E., Herz J., Tanowitz H.B. (2011). *Trypanosoma cruzi* utilizes the host low density lipoprotein receptor in invasion. PLoS Negl. Trop. Dis..

[40] Fernandes M.C., Cortez M., Geraldo Yoneyama K.A., Straus A.H., Yoshida N., Mortara R.A. (2007). Novel strategy in *Trypanosoma cruzi* cell invasion: Implication of cholesterol and host cell microdomains. Int. J. Parasitol..

[41] Torrecilhas A.C.T., Tonelli R.R., Pavanelli W.R., da Silva J.S., Schumacher R.I., de Souza W., e Silva N.C., Abrahamsohn I.d.A., Colli W., Alves M.J.M. (2009). *Trypanosoma cruzi*: Parasite shed vesicles increase heart parasitism and generate an intense inflammatory response. Microbes Infect..

[42] Garcia-Silva M.R., das Neves R.F.C., Cabrera-Cabrera F., Sanguinetti J., Medeiros L.C., Robello C., Naya H., Fernandez-Calero T., Souto-Padron T., de Souza W. (2014). Extracellular vesicles shed by *Trypanosoma cruzi* are linked to small RNA pathways, life cycle regulation, and susceptibility to infection of mammalian cells. Parasitol. Res..

[43] Mantel P.-Y., Marti M. (2014). The role of extracellular vesicles in Plasmodium and other protozoan parasites. Cell. Microbiol..

[44] Carrera-Bravo C., Koh E.Y., Tan K.S.W. (2021). The roles of parasite-derived extracellular vesicles in disease and host-parasite communication. Parasitol. Int..

[45] Ribeirão M., Pereira-Chioccola V.L., Rénia L., Augusto Fragata Filho A., Schenkman S., Rodrigues M.M. (2000). Chagasic patients develop a type 1 immune response to *Trypanosoma cruzi* trans-sialidase. Parasite Immunol..

[46] Campetella O., Buscaglia C.A., Mucci J., Leguizamón M.S. (2020). Parasite-host glycan interactions during *Trypanosoma cruzi* infection: Trans-Sialidase rides the show. Biochim. Biophys. Acta Mol. Basis Dis..

[47] Nardy A.F.F.R., Freire-de-Lima C.G., Pérez A.R., Morrot A. (2016). Role of *Trypanosoma cruzi* Trans-sialidase on the Escape from Host Immune Surveillance. Front. Microbiol..

[48] Schnapp A.R., Eickhoff C.S., Sizemore D., Curtiss R., Hoft D.F. (2002). Cruzipain induces both mucosal and systemic protection against *Trypanosoma cruzi* in mice. Infect. Immun..

[49] Guiñazú N., Pellegrini A., Giordanengo L., Aoki M.P., Rivarola H.W., Cano R., Rodrigues M.M., Gea S. (2004). Immune response to a major *Trypanosoma cruzi* antigen, cruzipain, is differentially modulated in C57BL/6 and BALB/c mice. Microbes Infect..

[50] Moreira L.R., Prescilla-Ledezma A., Cornet-Gomez A., Linares F., Jódar-Reyes A.B., Fernandez J., Vannucci A.K.I., De Pablos L.M., Osuna A. (2021). Biophysical and Biochemical Comparison of Extracellular Vesicles Produced by Infective and Non-Infective Stages of *Trypanosoma cruzi*. Int. J. Mol. Sci..

[51] Ishii T., Warabi E., Yanagawa T. (2012). Novel roles of peroxiredoxins in inflammation, cancer and innate immunity. J. Clin. Biochem. Nutr..

[52] Rhee S.G., Woo H.A. (2020). Multiple functions of 2-Cys peroxiredoxins, I and II, and their regulations via post-translational modifications. Free Radic. Biol. Med..

[53] Sun Y., Liu W.-Z., Liu T., Feng X., Yang N., Zhou H.-F. (2015). Signaling pathway of MAPK/ERK in cell proliferation, differentiation, migration, senescence and apoptosis. J. Recept. Signal Transduct. Res..

[54] Nkabyo Y.S., Go Y.-M., Ziegler T.R., Jones D.P. (2005). Extracellular cysteine/cystine redox regulates the p44/p42 MAPK pathway by metalloproteinase-dependent epidermal growth factor receptor signaling. Am. J. Physiol. Gastrointest. Liver Physiol..

[55] Baruch M., Hertzog B.B., Ravins M., Anand A., Youting C.C., Biswas D., Tirosh B., Hanski E. (2014). Induction of endoplasmic reticulum stress and unfolded protein response constitutes a pathogenic strategy of group A streptococcus. Front. Cell. Infect. Microbiol..

[56] Hu P., Han Z., Couvillon A.D., Exton J.H. (2004). Critical role of endogenous Akt/IAPs and MEK1/ERK pathways in counteracting endoplasmic reticulum stress-induced cell death. J. Biol. Chem..

[57] Kapadia S.B., Barth H., Baumert T., McKeating J.A., Chisari F.V. (2007). Initiation of hepatitis C virus infection is dependent on cholesterol and cooperativity between CD81 and scavenger receptor B type I. J. Virol..

[58] Feeney E.R., McAuley N., O’Halloran J.A., Rock C., Low J., Satchell C.S., Lambert J.S., Sheehan G.J., Mallon P.W.G. (2013). The expression of cholesterol metabolism genes in monocytes from HIV-infected subjects suggests intracellular cholesterol accumulation. J. Infect. Dis..

[59] Lafont F., Tran Van Nhieu G., Hanada K., Sansonetti P., van der Goot F.G. (2002). Initial steps of Shigella infection depend on the cholesterol/sphingolipid raft-mediated CD44-IpaB interaction. EMBO J..

[60] Cao F., Castrillo A., Tontonoz P., Re F., Byrne G.I. (2007). Chlamydia pneumoniae--induced macrophage foam cell formation is mediated by Toll-like receptor 2. Infect Immun..

[61] Pandey A.K., Sassetti C.M. (2008). Mycobacterial persistence requires the utilization of host cholesterol. Proc. Natl. Acad. Sci. USA.

[62] Robibaro B., Stedman T.T., Coppens I., Ngo H.M., Pypaert M., Bivona T., Nam H.W., Joiner K.A. (2002). *Toxoplasma gondii* Rab5 enhances cholesterol acquisition from host cells. Cell. Microbiol..

[63] D’avila H., Freire-De-Lima C.G., Roque N.R., Teixeira L., Barja-Fidalgo C., Silva A.R., Melo R.C.N., DosReis G.A., Castro-Faria-Neto H.C., Bozza P.T. (2011). Host cell lipid bodies triggered by *Trypanosoma cruzi* infection and enhanced by the uptake of apoptotic cells are associated with prostaglandin E₂ generation and increased parasite growth. J. Infect. Dis..

[64] Nishikawa Y., Ibrahim H.M., Kameyama K., Shiga I., Hiasa J., Xuan X. (2011). Host cholesterol synthesis contributes to growth of intracellular *Toxoplasma gondii* in macrophages. J. Vet. Med. Sci..

[65] Rabhi I., Rabhi S., Ben-Othman R., Rasche A., Daskalaki A., Trentin B., Piquemal D., Regnault B., Descoteaux A., Guizani-Tabbane L. (2012). Transcriptomic signature of *Leishmania* infected mice macrophages: A metabolic point of view. PLoS Negl. Trop. Dis..

[66] Kulkarni R., Wiemer E.A.C., Chang W. (2021). Role of Lipid Rafts in Pathogen-Host Interaction—A Mini Review. Front. Immunol..

[67] Pike L.J. (2003). Lipid rafts: Bringing order to chaos. J. Lipid Res..

[68] Liendo A., Visbal G., Piras M.M., Piras R., Urbina J.A. (1999). Sterol composition and biosynthesis in *Trypanosoma cruzi* amastigotes. Mol. Biochem. Parasitol..

[69] Korn E.D., Von Brand T., Tobie E.J. (1969). The sterols of *Trypanosoma cruzi* and *Crithidia fasciculata*. Comp. Biochem. Physiol..

[70] Mauricio D., Castelblanco E., Alonso N. (2020). Cholesterol and Inflammation in Atherosclerosis: An Immune-Metabolic Hypothesis. Nutrients.

[71] Sunnemark D., Harris R.A., Frostegård J., Orn A. (2000). Induction of early atherosclerosis in CBA/J mice by combination of *Trypanosoma cruzi* infection and a high cholesterol diet. Atherosclerosis.

[72] Houston-Ludlam G.A., Belew A.T., El-Sayed N.M. (2016). Comparative Transcriptome Profiling of Human Foreskin Fibroblasts Infected with the Sylvio and Y Strains of *Trypanosoma cruzi*. PLoS ONE.

[73] Bonney K.M., Engman D.M. (2015). Autoimmune pathogenesis of Chagas heart disease: Looking back, looking ahead. Am. J. Pathol..

[74] Soares M.B., Pontes-De-Carvalho L., Ribeiro-Dos-Santos R. (2001). The pathogenesis of Chagas’ disease: When autoimmune and parasite-specific immune responses meet. An. Acad. Bras. Cienc..

[75] Sasu S., LaVerda D., Qureshi N., Golenbock D.T., Beasley D. (2001). Chlamydia pneumoniae and Chlamydial Heat Shock Protein 60 Stimulate Proliferation of Human Vascular Smooth Muscle Cells via Toll-like Receptor 4 and p44/p42 Mitogen-Activated Protein Kinase Activation. Circ. Res..

[76] Kimura R., Komaki-Yasuda K., Kawazu S.-I., Kano S. (2013). 2-Cys peroxiredoxin of *Plasmodium falciparum* is involved in resistance to heat stress of the parasite. Parasitol. Int..

[77] Teixeira F., Castro H., Cruz T., Tse E., Koldewey P., Southworth D.R., Tomás A.M., Jakob U. (2015). Mitochondrial peroxiredoxin functions as crucial chaperone reservoir in *Leishmania infantum*. Proc. Natl. Acad. Sci. USA.

[78] Garcia-Silva M.R., Cabrera-Cabrera F., das Neves R.F.C., Souto-Padrón T., de Souza W., Cayota A. (2014). Gene expression changes induced by *Trypanosoma cruzi* shed microvesicles in mammalian host cells: Relevance of tRNA-derived halves. Biomed. Res. Int..

